# Regulator of G-Protein Signaling-4 Attenuates Cardiac Adverse Remodeling and Neuronal Norepinephrine Release-Promoting Free Fatty Acid Receptor FFAR3 Signaling

**DOI:** 10.3390/ijms23105803

**Published:** 2022-05-22

**Authors:** Alexandra M. Carbone, Jordana I. Borges, Malka S. Suster, Anastasiya Sizova, Natalie Cora, Victoria L. Desimine, Anastasios Lymperopoulos

**Affiliations:** Laboratory for the Study of Neurohormonal Control of the Circulation, Department of Pharmaceutical Sciences, Nova Southeastern University College of Pharmacy, Fort Lauderdale, FL 33328-2018, USA; ac3411@mynsu.nova.edu (A.M.C.); jb3837@mynsu.nova.edu (J.I.B.); ms5019@mynsu.nova.edu (M.S.S.); as4532@mynsu.nova.edu (A.S.); nc1174@mynsu.nova.edu (N.C.); victoria.desimine@va.gov (V.L.D.)

**Keywords:** G-protein-coupled receptor, signal transduction, FFAR3, RGS4, catecholamine, cardiomyocyte, sympathetic neuron, norepinephrine, inflammation, propionic acid

## Abstract

Propionic acid is a cell nutrient but also a stimulus for cellular signaling. Free fatty acid receptor (FFAR)-3, also known as GPR41, is a Gi/o protein-coupled receptor (GPCR) that mediates some of the propionate’s actions in cells, such as inflammation, fibrosis, and increased firing/norepinephrine release from peripheral sympathetic neurons. The regulator of G-protein Signaling (RGS)-4 inactivates (terminates) both Gi/o- and Gq-protein signaling and, in the heart, protects against atrial fibrillation via calcium signaling attenuation. RGS4 activity is stimulated by β-adrenergic receptors (ARs) via protein kinase A (PKA)-dependent phosphorylation. Herein, we examined whether RGS4 modulates cardiac FFAR3 signaling/function. We report that RGS4 is essential for dampening of FFAR3 signaling in H9c2 cardiomyocytes, since siRNA-mediated RGS4 depletion significantly enhanced propionate-dependent cAMP lowering, Gi/o activation, p38 MAPK activation, pro-inflammatory interleukin (IL)-1β and IL-6 production, and pro-fibrotic transforming growth factor (TGF)-β synthesis. Additionally, catecholamine pretreatment blocked propionic acid/FFAR3 signaling via PKA-dependent activation of RGS4 in H9c2 cardiomyocytes. Finally, RGS4 opposes FFAR3-dependent norepinephrine release from sympathetic-like neurons (differentiated Neuro-2a cells) co-cultured with H9c2 cardiomyocytes, thereby preserving the functional βAR number of the cardiomyocytes. In conclusion, RGS4 appears essential for propionate/FFAR3 signaling attenuation in both cardiomyocytes and sympathetic neurons, leading to cardioprotection against inflammation/adverse remodeling and to sympatholysis, respectively.

## 1. Introduction

The short-chain free fatty acid (SCFA) propionate stimulates cellular signaling primarily via two different G-protein-coupled receptors (GPCRs) and free fatty acid receptor (FFAR)-2 and -3 [1,2,3]. FFAR3, also known as GPR41, regulates cardiovascular function via effects in peripheral sympathetic neurons, wherein it promotes neuronal firing and norepinephrine (NE) synthesis and release [4,5]. FFAR3 couples to Gi/o proteins, which can activate phospholipase (PLC)-β_2/3_ and downstream calcium signaling via their free Gβγ subunits [4]. Synapsin-2b is ultimately phosphorylated and activated to induce vesicle fusion with the neuronal plasma membrane and NE exocytosis/synaptic release from sympathetic nerve endings [5]. In addition, the stimulatory effect of propionate/FFAR3 in sympathetic neurons is directly regulated by β-adrenergic receptors (ARs), since FFAR3 seems to upregulate βAR function in sympathetic ganglia [4].

The regulator of G-protein Signaling (RGS)-4 is highly expressed in the heart and brain [6,7,8,9,10]. In fact, brain RGS4 has long been a drug development target for schizophrenia [6,7]. It belongs to the B/R4 family of RGS proteins and inactivates Gi/o- and G_q/11_ protein signaling via direct interactions with the Gα subunits of these heterotrimeric G protein families, whose guanosine triphosphatase (GTPase) activities it dramatically augments [6,7,8,9,10,11]. Importantly, RGS4 has been reported, uniquely among RGS proteins, to also directly bind Gi/o-derived free G_βγ_ subunits and PLCβ, thereby blocking PLCβ activation and downstream calcium signaling independently of its GAP (GTPase activating protein) action on the Gα subunits [12,13]. RGS4 is abundantly expressed in the sinoatrial (SA) and atrioventricular (AV) nodal regions of the heart, as well as throughout the atria [14,15]. Exogenous overexpression of RGS4 in cardiomyocytes attenuates endothelin receptor signaling, reducing phospholipase C (PLC)-β activation, contractility in the long term, and cardiac hypertrophy [16,17,18]. Indeed, RGS4 ameliorates cardiac hypertrophy induced by pressure overload via direct inhibition of the Gq protein-dependent pro-hypertrophic signaling in murine hearts [16,17,18]. RGS4 is also upregulated in rat hypertrophic hearts [19] and, importantly, in human failing hearts from both acute and end-stage chronic heart failure patients [20,21]. Moreover, RGS4 protects against abnormal calcium transients and signaling that can lead to tachyarrhythmias and atrial fibrillation (AFib) [10,22,23]. Taken together, these studies suggest a cardioprotective role for RGS4 in the heart and its upregulation in pressure, overload-induced hypertrophy, and in human heart failure, they may reflect an adaptive mechanism to compensate for compromised function and for self-defense against toxic insults that increase myocardial oxygen demand.

FFAR3 is expressed in various tissues including in the heart [1,2,3] and is known to promote tissue inflammation and, as mentioned above, sympathetic nervous system (SNS) activity, neuronal firing, and NE release [1,2,3]. The latter is cardiotoxic, especially for the failing heart, as it increases myocardial oxygen/metabolic demand [24,25]. However, very little (virtually nothing) is currently known regarding cardiac FFAR3 signaling, per se. Propionic acid has been reported to alleviate cardiac dysfunction in Akt2-knockout mice [26], but that study did not examine whether its actions were FFAR3- or FFAR2-mediated (or both). Since FFAR3 couples to G_i/o_ proteins, however, its signaling can very well be a candidate for RGS4-mediated regulation. In the present study, we sought to investigate whether RGS4 indeed regulates FFAR3 signaling in cardiac myocytes, which could underlie part of the cardioprotective actions of RGS4. We report that RGS4 inhibits propionic acid/FFAR3-induced G_i/o_ protein signaling in H9c2 cardiomyocytes, mitigating the pro-inflammatory and pro-fibrotic signaling of FFAR3. Additionally, cardiac βARs stimulate RGS4 to impede cardiac FFAR3 signaling. Finally, RGS4 also opposes FFAR3-dependent NE release from cultured sympathetic neurons, thereby preserving the βAR function of co-cultured H9c2 cardiomyocytes.

## 2. Results

### 2.1. RGS4 Inactivates Cardiac FFAR3-Stimulated Gi/O Protein Signaling

**Figure 1:**
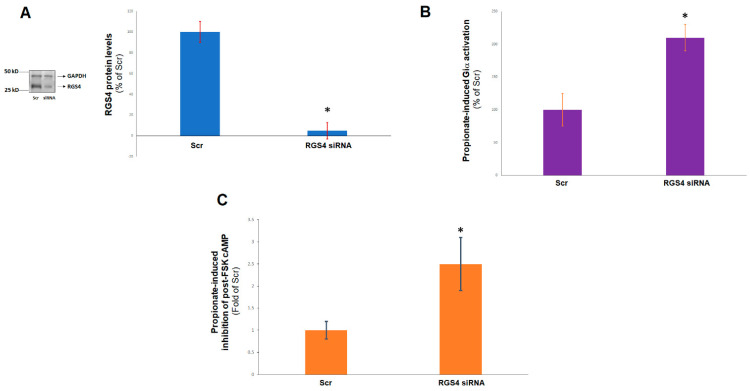
RGS4 and FFAR3-dependent Gi/o protein activation in cardiac myocytes. (**A**) Immunoblotting for RGS4 to confirm the efficiency of the siRNA-mediated knockdown of RGS4 in H9c2 cells at 48 hrs post-siRNA transfection. A representative blot, including for glyceraldehyde 3′-phosphate dehydrogenase (GAPDH) as a loading control, is shown on the left, and the % protein reduction, based on densitometric analysis of 4 independent experiments, is shown on the right. Scr: Scrambled siRNA (control); *, *p* < 0.05; *n* = 4 independent experiments per condition. (**B**) A 1 mM propionic acid-induced Giα activation in control (scrambled siRNA-transfected, Scr), or in RGS4-depleted (RGS4 siRNA) H9c2 cells. *, *p* < 0.05; *n* = 4 independent experiments. (**C**) A 1 mM propionic acid-mediated inhibition of 10 μM forskolin (FSK)-induced cAMP accumulation in the same cells, expressed as a % of the inhibition observed in the control (Scr) H9c2 cardiac cells. Forskolin alone induced similar levels of cAMP accumulation in both cell clones (data not shown). *, *p* < 0.05; *n* = 4 independent experiments.

Since FFAR3 signals through Gi/o proteins, which are known to be inactivated by RGS4, we sought to explore whether RGS4 regulates FFAR3 signaling in cardiac myocytes. To that end, we knocked down RGS4 via siRNA in H9c2 cardiomyocytes and compared the responses of the RGS4-depleted cells with those of control cells to propionic acid, the prototypic SCFA agonist for FFAR3 [3]. After confirming a robust (>90%) RGS4 protein knockdown induced by the siRNA treatment (Figure 1A), we measured propionate-induced Giα subunit activity in the two cell clones: control (receiving scrambled siRNA) and RGS4-depleted (transfected with rat RGS4-specific siRNA) H9c2 cardiac cells. We found that RGS4 siRNA-mediated depletion led to a markedly higher Giα activation, as measured by guanosine triphosphate (GTP) binding, in response to acute propionic acid stimulation (Figure 1B). Consistent with this, inhibition of forskolin-stimulated synthesis of the second messenger cyclic 3′, 5′-adenosine monophosphate (cAMP) was also significantly enhanced by propionate in RGS4-depleted cardiomyocytes (Figure 1C). Taken together, these results suggest that indeed, RGS4 directly blocks Gi/o protein signaling stimulated by propionic acid via FFAR3 in H9c2 cardiac myocytes.

### 2.2. RGS4 Opposes Cardiac FFAR3 Pro-Inflammatory and Pro-Fibrotic Signaling 

**Figure 2:**
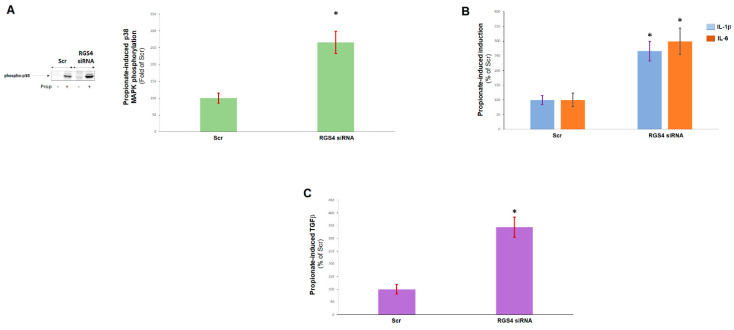
RGS4 and FFAR3 signaling in cardiac myocytes. (**A**) A 1 mM propionate-induced p38 MAPK phosphorylation/activation in the control (scrambled siRNA-transfected, Scr) or in RGS4-depleted (RGS4 siRNA) H9c2 cells. A representative blot is shown on the left and the densitometric quantitation on the right. Prop: Propionate. *, *p* < 0.05; *n* = 3 independent experiments performed in triplicate. (**B**,**C**) A 1 mM propionate-induced IL-1β and IL-6 (**B**) or TGF-β_1_ (**C**) protein synthesis in these cells. *, *p* < 0.05, vs. respective Scr; *n* = 3 independent measurements per condition per cell clone.

Next, we examined the functional consequences of RGS4-mediated inhibition of FFAR3 Gi/o protein signaling in cardiac myocytes. RGS4 depletion resulted in markedly enhanced activation of the pro-inflammatory p38 mitogen-activated protein kinase (MAPK) in response to propionic acid stimulation in H9c2 cardiomyocytes (Figure 2A). Additionally, propionic acid-induced interleukin (IL)-1β and IL-6 synthesis and secretion were also increased in RGS4-knocked-down cardiomyocytes (Figure 2B), and the same was observed for propionic acid-dependent production of transforming growth factor (TGF)-β_1_, a major pro-fibrotic stimulus [27] (Figure 2C). Thus, it appears that propionate/FFAR3 promote cardiac inflammation and fibrosis, two major cardiac-adverse, remodeling-related processes, and this is blocked by RGS4 via FFAR3-coupled Gi/o protein inactivation in cardiac myocytes. 

### 2.3. β ARs Reduce Cardiac FFAR3 Signaling via PKA-Dependent RGS4 Stimulation

**Figure 3:**
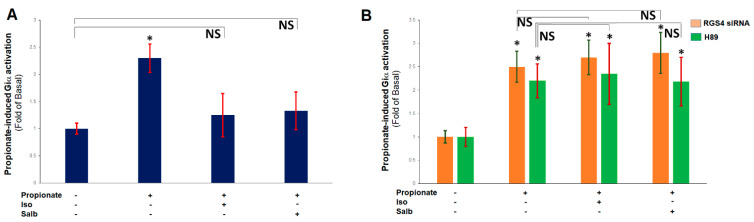
βARs negatively regulate FFAR3 signaling via RGS4 and PKA in cardiac myocytes. (**A**) Giα activation in native H9c2 cells treated with 1 mM propionate, 10 μM isoproterenol (Iso), 10 μM salbutamol (Salb), or a combination thereof. *, *p* < 0.05, vs. basal (no treatment); NS: Not significant (vs. basal-No treatment) at *p* = 0.05; *n* = 3 independent measurements (in duplicate) per condition. (**B**) Giα activation in RGS4-depleted (RGS4 siRNA) or in native H9c2 cells pre-treated with 10 μM H89 (H89), in response to 1 mM propionate alone, or to 1 mM propionate in the presence of 10 μM isoproterenol (Iso) or 10 μM salbutamol (Salb). *, *p* < 0.05, vs. respective basal (no treatment); NS: Not significant (vs. respective propionate alone) at *p* = 0.05; *n* = 3 independent determinations (in duplicate) per condition per cell clone.

Protein kinase A (PKA), the major effector of cAMP, has been reported to directly phosphorylate and activate RGS4, by inducing its plasma membrane translocation, in cultured smooth muscle cells, with Ser52 of RGS4, being one of the major phosphorylation sites [13]. Since βARs activate PKA via coupling to Gs proteins, whose Gα subunits stimulate adenylyl cyclase-mediated cAMP synthesis [26,27], we next examined whether PKA activates RGS4 also in cardiomyocytes, thereby mediating a βAR-FFAR3 crosstalk in the heart. Indeed, pretreatment of H9c2 cardiomyocytes with either isoproterenol, which stimulates both β_1_- and β_2_ARs, or salbutamol (albuterol), which is a β_2_AR-selective agonist [27,28,29], markedly reduced propionic acid-elicited Giα activity in control and wild-type H9c2 cardiomyocytes (Figure 3A), but both catecholamines failed to do so in RGS4-depleted cells (Figure 3B). Furthermore, in the presence of H89, a well-characterized PKA inhibitor [30], neither isoproterenol nor salbutamol, had any detectable effect on propionic acid-dependent Giα activation in the native, wild-type H9c2 cardiomyocytes (Figure 3B). Taken together, these results strongly suggest that catecholamines via both β_1_- and β_2_ARs induce PKA-dependent activation of RGS4, which subsequently impedes FFAR3 signaling in cardiac myocytes.

### 2.4. RGS4 Inhibits FFAR3-Stimulated NE Release from Sympathetic Neurons

**Figure 4:**
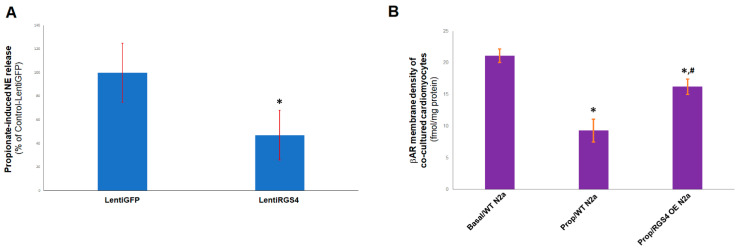
RGS4 and neuronal FFAR3-mediated NE release. (**A**) Levels of NE released into the culture medium from the control (infected with a lentivirus encoding for green fluorescent protein, LentiGFP) or RGS4-overexpressing (infected with a lentivirus encoding for full length RGS4, LentiRGS4) differentiated Neuro-2A (N2a) cells in response to 1 mM of propionate stimulation. No statistically significant difference in NE release was observed between the two cell clones under basal conditions, i.e., without propionate stimulation (data not shown). *, *p* < 0.05; *n* = 5 independent measurements per condition per cell clone. (**B**) βAR density (B_max_) in plasma membranes isolated from H9c2 cardiomyocytes at 24 hrs post-stimulation with 1 mM propionate (Prop) or vehicle (Basal) of co-cultured control, wild-type (WT) or RGS4-overexpressing (RGS4 OE) N2a cells. The βAR density measured with vehicle-treated RGS4-overexpressing N2a cells (Basal/RGS4 OE N2a) was similar to the one calculated with Basal/WT N2a cells (not shown). *, *p* < 0.05, vs. Basal/WT N2a; **^#^**, *p* < 0.05 vs. Prop/WT N2a; *n* = 3 independent determinations per condition per cell clone.

Since FFAR3 is also involved in sympathetic neuronal activation stimulating NE release [4], we also checked for a potential regulatory role of RGS4 in cardiac sympathetic neuronal FFAR3 signaling. To mimic the in vivo situation of cardiac sympathetic nerve terminals releasing NE directly into cardiac myocytes, we co-cultured H9c2 cardiomyocytes together with nerve growth factor (NGF)-differentiated mouse Neuro-2A cells, known to synthesize and release NE (as well as dopamine), thus, functioning, in essence, like sympathetic neurons [4,31]. Given that endogenous FFAR3 expression in Neuro-2A cells is equivocal (very low at best) [4], we transfected these neuroblastoma cells to express human FFAR3, prior to their NGF-elicited neuronal differentiation. Propionate treatment led to significant NE release from the FFAR3-expressing differentiated Neuro-2A cells, which was partially blocked by RGS4 lentiviral-mediated overexpression (Figure 4A). We then co-cultured RGS4-overexpressing or control neuronal cells together with (native, wild-type) H9c2 cardiac myocytes, and, 24 h post-propionic acid treatment, we measured the βAR membrane density of the H9c2 cardiac cells. Propionate treatment of the control neuronal cells led to a significant βAR agonist-dependent downregulation in the co-cultured cardiomyocytes, as expected due to the propionate/FFAR3-elicited NE release into the cell co-culture medium (Figure 4B). Specifically, normal (basal) total βAR membrane density of H9c2 cardiomyocytes was estimated at 21.1 ± 1.05 fmol/mg of protein and it was reduced to 9.3 ± 1.8 fmol/mg of protein upon propionate stimulation of co-cultured control Neuro-2A cells (*n* = 4 independent determinations per condition) (Figure 4B). However, cardiomyocyte βAR membrane density was substantially higher 24 h post-propionate challenge, when co-cultured with RGS4-overexpressing neuronal cells (16.2 ± 1.2 fmol/mg of protein, *n* = 4; Figure 4B). That was presumably because of the lower NE release from the RGS4-overexpressing neuronal cells, which reduced the levels of NE activating the H9c2 cardiomyocyte-residing βARs, causing their homologous downregulation. Taken together, these findings indicate that RGS4 opposes propionate/FFAR3 signaling also in sympathetic neurons, which may lower neuronal activity and NE release, thereby largely preserving βAR density/function of the innervated myocardium. 

## 3. Discussion

**Figure 5:**
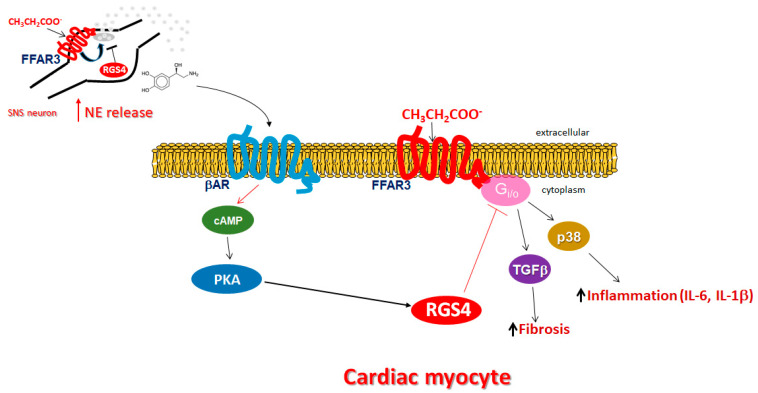
Schematic illustration of propionic acid-activated FFAR3 signaling and function in cardiac myocytes and in sympathetic neurons, and the inhibitory role of RGS4 in both tissues. See text for details. SNS: Sympathetic nervous system.

In the present study, we have uncovered an important regulatory role for RGS4 at keeping propionic acid signaling through FFAR3/GPR41 in check in both cardiomyocytes and sympathetic neurons (Figure 5). On the one hand, myocardial FFAR3 activated by SCFAs, such as propionic acid, upregulates several pro-inflammatory (IL-1β, IL-6, activated p38 MAPK) [32] and pro-fibrotic (e.g., TGFβ [26]) factors, which is highly likely to lead to exacerbated adverse remodeling in the presence of a cardiac insult, such as myocardial ischemia/infarction or pressure overload/severe hypertension (Figure 5). Furthermore, TGFβ, but also inflammation and fibrosis in general, have been implicated in cardiac aging [33,34,35]. Thus, FFAR3 signaling may also promote cardiac aging and RGS4, and by opposing FFAR3 effects, may have anti-aging properties in the heart. On the other hand, FFAR3 present in post-ganglionic sympathetic neurons, including cardiac sympathetic nerve terminals, promotes neuronal firing and activity resulting in elevated NE release [4] (Figure 5). RGS4 terminates FFAR3 Gi/o protein-dependent signaling in both cell types (cardiomyocytes and sympathetic neurons), thereby lowering propionic acid-dependent cardiac inflammation/fibrosis and sympathetic activity/NE release at the same time (Figure 5). The latter is particularly useful in the setting of chronic heart failure, during which the failing heart needs to be protected against the toxicity of elevated catecholamine levels [36,37,38].

Indeed, we found that inhibition of neuronal FFAR3-dependent NE release by RGS4 markedly reduced agonist (NE)-dependent downregulation of βARs in co-cultured cardiomyocytes, which suggests that RGS4 in sympathetic neurons has the potential of preserving, at least in part, the adrenergic and inotropic reserves of the failing heart. This is also consistent with the old studies showing a cardioprotective effect of RGS4 against pressure overload (increased afterload)-induced hypertrophy [16,18]. Indeed, apart from shielding the heart from pathological Gq protein-dependent pro-hypertrophic signaling that diminishes cardiac function [17,18], cardiac RGS4 overexpression was shown in that 1999 study [16] to preserve the positive inotropic response of transgenic mice to the catecholaminergic agonist dobutamine post-pressure overload. Taken together, these findings strongly suggest that RGS4, in both the heart and the sympathetic nervous system, may serve as an important molecular brake for the cardiotoxic effects of propionic acid/FFAR3 signaling. Therefore, systemic RGS4 pharmacological stimulation might be of therapeutic value in heart disease, particularly in chronic human heart failure and in systemic hypertension.

Our present study expands on the already established cardioprotective role of RGS4, which is upregulated, most likely as a compensatory mechanism, in the hearts of experimental animals of cardiac hypertrophy secondary to aortic or pulmonary artery banding in vivo [19] and in human failing hearts explanted from dilated or ischemic cardiomyopathy patients, or from end-stage or acute human heart failure patients [10,20,21]. Additionally, RGS4 exerts crucial cardioprotective effects in the atrial myocardium against AFib pathogenesis and pro-arrhythmogenic Ca^2+^ transient generation [9,10,22,23].

In contrast to its well-documented actions in the myocardium, little (if anything at all) is known about the effects of RGS4 in sympathetic neurons. FFAR3 is robustly expressed in murine peripheral sympathetic neurons, including cardiac sympathetic nerve terminals, wherein it regulates not only cell metabolism but also neuronal activity via stimulation of NE release [4] (Figure 5). Although both NE and epinephrine mediate the effects of the sympathetic nervous system on all cells and tissues, NE is the neurotransmitter synthesized, stored, and released from sympathetic neurons, whereas epinephrine is a hormone synthesized in the adrenal medulla and secreted into the systemic circulation [39,40,41]. FFAR3 knockout mice display significantly lower catecholamine synthesis, as evidenced by tyrosine hydroxylase downregulation, the enzyme that catalyzes the rate-limiting step of catecholamine biosynthesis [42], as well as lower sympathetic neuronal firing rate and heart rate [1,4]. 

Mechanistically, FFAR3 stimulates NE release via Gi/o-derived free Gβγ subunit activation of PLCβ_2/3_ [4]. PLCβ_2/3_, in turn, induces Ca^2+^ signaling that ultimately results in activation of extracellular signal-regulated kinase (ERK)1/2, which then has phosphorylate synapsin-2b at Ser426 to induce vesicle fusion with the neuronal plasma membrane and subsequent NE release from sympathetic nerve endings [5]. RGS4 is known to directly bind not only Gα subunits (which it deactivates via its RGS function), but also Gi/o-derived free Gβγ subunits and PLCβ, thereby inhibiting PLCβ activation directly (independently of its RGS function) [12,13]. It is thus tempting to speculate that direct PLCβ binding and inhibition is the mechanism underlying RGS4’s inhibitory effect on neuronal FFAR3 signaling towards NE release (and perhaps also some of its effects on cardiac FFAR3 signaling). However, this awaits verification in future studies and efforts to delineate the full signaling pathway(s) underlying the effects of RGS4 on cardiac and neuronal FFAR3 signaling are under way in our laboratory.

Finally, we have uncovered an important RGS4-centric regulatory negative feedback loop operating in cardiac myocytes between βARs (in particular, β_2_AR) and FFAR3: βAR-activated PKA phosphorylates and stimulates RGS4 to abrogate SCFA/FFAR3 signaling and function in cardiac myocytes (Figure 5). At the same time, FFAR3-activated Giα inhibits adenylyl cyclase and thus, cAMP synthesis, reducing PKA activation, which would indirectly decrease βAR-dependent RGS4 activity and enhance FFAR3 signaling. However, this may not be the case given a very recent study that suggests the existence of receptor-associated independent cAMP nanodomains operating as individual, independent cell signaling units inside the same cell; in other words, a GPCR may not interfere with the cAMP pool associated with a different receptor, despite residing at the same plasma membrane and perhaps even in close proximity with one another [43]. It should also be noted here that RGS4, like all other canonical RGS proteins identified to date, does not directly affect (i.e., inactivates) Gsα subunits, to which βARs normally couple [11]. The presence of this RGS4-mediated regulatory crosstalk between βARs and FFAR3 is entirely consistent with the finding by Kimura et al. that the effect of propionate/FFAR3 on heart rate was suppressed by pretreatment with the non-subtype selective β-blocker propranolol, which suggested reciprocal regulation of FFAR3 signaling by βARs [4]. It is also in line with the role of PKA in PLCβ blockade, (in part) via RGS4 activation, in gastric smooth muscle cells [13]. Thus, it seems that PKA activates RGS4 via phosphorylation in the heart, as well. Moreover, since β_2_AR-activated RGS4 obstructs cardiac FFAR3 pro-inflammatory and pro-fibrotic signaling (Figure 5), these findings are also consistent with a potential beneficial role for the cardiac β_2_AR subtype in cardiac reverse remodeling [26,31], in direct juxtaposition to the much more abundant β_1_AR subtype [28,36,44]. Of note, in addition to the cardiac βAR–FFAR3 crosstalk via RGS4, a similar negative feedback crosstalk also exists between sympathetic neuronal FFAR3 and cardiac βARs (Figure 5). More specifically, neuronal FFAR3 increases the amount of NE that activates cardiac βARs, promoting βAR homologous desensitization and downregulation in the myocardium that, over time, diminishes cardiac βAR function and number [36]. RGS4 again opposes neuronal FFAR3-dependent NE release, thereby helping to counteract βAR homologous desensitization/downregulation and to preserve myocardial βAR function (Figure 5).

The present study has two major limitations: (a) Although it was done in physiologically relevant (cardiomyocyte-like and sympathetic neuron-like) cells, these cells were not, nonetheless, primary bona fide differentiated cells; and (b) Our findings obviously require confirmation in in vivo models and settings. Nevertheless, given that the cell lines we employed closely mimicked primary, differentiated cell cultures and were still physiologically relevant, our present findings are quite likely to hold true in the heart and in cardiac sympathetic neurons in vivo, as well. 

In summary, we report here, for the first time to our knowledge, that: (a) Cardiac FFAR3 promotes inflammation and fibrosis via its classic Gi/o protein signaling, which is opposed by RGS4; (b) Catecholamines negatively modulate cardiac FFAR3 signaling via PKA-mediated RGS4 activation; and (c) RGS4 dampens FFAR3 signaling towards NE release in sympathetic neurons, thereby potentially affording sympatholysis that can help preserve cardiac βAR function, especially in the setting of chronic heart failure. Cardiac, as well as neuronal, RGS4 stimulation might, thus, be advantageous in helping the failing heart cope with the toxic effects, both direct and indirect (via sympathetic hyperactivation), of SCFAs that are aberrantly upregulated in human heart failure [45,46]. If confirmed in vivo, these findings will expand the growing list of beneficial effects of RGS4 in the myocardium, which already includes keeping heart rate and post-pressure overload cardiac hypertrophy in check. Although the focus of RGS protein-targeted drug discovery efforts has been almost exclusively on development of inhibitors, stimulation of specific RGS proteins is therapeutically desirable for certain conditions [10] and pharmacologically feasible, at least indirectly, by targeting RGS protein expression (i.e., inhibiting degradation/increasing stability of the RGS protein) [7]. Our present study provides substantial evidence that, in the case of cardiac FFAR3 signaling, development of an RGS4 activator or expression enhancer may be worth pursuing.

## 4. Materials and Methods

### 4.1. Materials

All chemicals and pharmacological agents, including propionic acid, forskolin, isoproterenol, and salbutamol, were from Sigma-Aldrich (St. Louis, MO, USA).

### 4.2. Cell Culture, Transfections, and Treatments

The H9c2 rat cardiomyoblast and Neuro-2A mouse neuroblastoma cell lines were purchased from American Type Culture Collection (Manassas, VA, USA) and cultured as previously described [4,47]. For siRNA-mediated knockdown of RGS4 in H9c2 cells, an siRNA against rat RGS4 [Target RefSeqID: NM_017214, *Rgs4* gene of the Rattus norvegicus species; Sequences: CCAAAUAUUGAUCUGUAUUdTdT (Sense); AAUACAGAUCAAUAUUUGGdTdT (Antisense)] was designed and synthesized (Sigma-Aldrich), along with a negative control (scrambled siRNA oligo, Cat. #SIC001; also from Sigma-Aldrich). Cells were transfected with either the RGS4-specific siRNA or the negative control (Scrambled) using the Mission^®^ siRNA transfection reagent (Cat. #S1452; Sigma-Aldrich) and 48 h later, Western blotting for RGS4 protein levels was performed to ascertain the efficiency of the knockdown. For RGS4 overexpression in Neuro-2A cells, the cells were first transfected (via the Lipofectamine^®^ 3000 Transfection Reagent method; ThermoFisher Scientific, Waltham, MA, USA) with a human FFAR3-encoding cDNA (CloneID #OHu24442, RefSeq #NM_005304.4; GenScript Biotech, Piscataway, NJ, USA) to overexpress FFAR3, and then differentiated into sympathetic-like neurons via daily treatments with 50 ng/mL NGF (Sigma-Aldrich) for 5 consecutive days [48]. Subsequently, infections of the differentiated Neuro-2A cells were performed with either a lentiviral construct encoding for full-length RGS4 (Lenti-CMV-RGS4v1, Cat. #LH848503; Vigene Biosciences/Charles River Laboratories, Rockville, MD, USA) to overexpress RGS4, or a control construct encoding for GFP (Cat. #CV10002; also from Charles River Laboratories). RGS4 overexpression was verified via Western blotting 48 hrs post-infection. For the Giα activity assay, plasma membranes were prepared from the H9c2 cells, and the ligands/agonists were added directly into the assay’s well plate immediately prior to the addition of the membrane suspension. For cAMP accumulation and p38 MAPK activation determinations, cells were harvested 30 min after agent/drug application, whereas for the ELISA measurements of IL-1β, Il-6, and TGFβ, cells were harvested 12 h post-treatment. Finally, NE release was measured 6 h post-propionate treatment, while H9c2 cardiomyocyte βAR density was assayed 24 h (the next day) post-propionic acid application to the H9c2-N2a cell co-culture.

### 4.3. Giα Activity Assay and cAMP Accumulation Determination

Giα activation was determined on isolated plasma membranes from H9c2 cells using a HTRF (homogeneous time-resolved fluorescence)-based GTP Gi binding assay kit (Cat. #62GTPPET; Cisbio-PerkinElmer, Inc., Waltham, MA, USA) and following the manufacturer’s protocols/instructions [49]. In essence, this assay measures the level of Giα activation following agonist stimulation of a GPCR, thanks to the use of a europium-labeled GTP analog (Eu-GTP). During the activation process of the heterotrimeric Gi protein, GDP dissociates from the Giα subunit and gets replaced by the assay’s fluorescent Eu-GTP analog that now binds to the Giα subunit, as the latter goes from its inactive to its active state. The fluorescent signal emitted by the Giα-bound Eu-GTP analog is detected via addition of an anti-Giα antibody, with the fluorescence intensity levels measured being directly proportional to the level of Giα activity. cAMP accumulation was measured with the “Direct cAMP ELISA kit” (Cat. #ADI-900-066; Enzo Life Sciences, Farmingdale, NY, USA), as described previously [27].

### 4.4. ELISA and Western Blotting

H9c2 cell extracts were prepared, as described previously [47], in a 20 mM Tris pH 7.4 buffer containing 137 mM of NaCl, 1% Nonidet P-40, 20% glycerol, 10 mM PMSF, 1 mM Na_3_VO_4_, 10 mM NaF, 2.5 mg/mL aprotinin, and 2.5 mg/mL leupeptin. Protein concentration was determined and equal amounts of protein per sample were used for ELISA or Western blotting. ELISA determinations for rat IL-1β (Cat. #EA-3005), rat IL-6 (Cat. #EA-3002), and rat TGFβ (Cat. #EA-3016) were done using kits from Signosis, Inc. (Santa Clara, CA, USA). NE levels released into cell culture medium/supernatant were also determined via ELISA, using the Alpco Ltd. Bi-CAT (Epinephrine and Norepinephrine) ELISA kit (Cat. #50-751-3487; ThermoFisher Scientific). RGS4 protein levels and p38 MAPK phosphorylation/activation were measured via Western blotting with antibodies against rat RGS4 (Cat. #9195; Cell Signaling Technology, Danvers, MA, USA), coupled with immunoblotting for GAPDH, as loading control, with an anti-GAPDH antibody (Cat. #sc-25778; Santa Cruz Biotechnology, Santa Cruz, CA, USA), and against phospho-p38 MAPK (Cat. #4511; Cell Signaling Technology). Immunoblots were revealed by enhanced chemiluminescence (ECL, Life Technologies, Grand Island, NY, USA) and visualized in a FluorChem E Digital Darkroom (Protein Simple, San Jose, CA, USA), as described previously [27]. Densitometry was performed with the AlphaView software (Protein Simple) in the linear range of signal detection (on non-saturated bands). 

### 4.5. βAR Density Measurements

βAR density was measured in isolated plasma membranes using ^125^I-CYP (Iodocyanopindolol; PerkinElmer, Inc.), as described [37]. Briefly, at 24 h post-propionate application, H9c2 cells were harvested, and plasma membrane fractions were prepared via lysis in a hypotonic buffer containing Tris^.^Cl (pH 7.5), EDTA (pH 8), EGTA, and protease inhibitors, followed by ultracentrifugation (62,000 rpm for 1 h at 4 °C). Protein content was determined and then incubations with ^125^I-CYP were performed in the presence of 80 μM alprenolol (for non-specific binding determination). The reactions were terminated in a cell harvester, followed by sample loading onto a gamma counter (Beckman Coulter, Brea, CA, USA) for radioactivity counts determination. Receptor densities are expressed as fmol/mg of membrane protein. 

### 4.6. Statistical Analysis

Data are generally expressed as mean ± SEM. Unpaired 2-tailed Student’s *t* test and one- or two-way ANOVA with Bonferroni test were generally performed for statistical comparisons, unless otherwise indicated. For most 3-group statistical comparisons, Dunnett’s test using SAS version 9 software (Cary, NC, USA) was used, as well. For all tests, a *p* value of <0.05 was generally considered to be significant.

## Data Availability

All data presented in this paper are available upon request to the correspondence author.
